# Telerehabilitation in the Treatment of Frozen Shoulder: A Case Report

**DOI:** 10.5195/ijt.2019.6288

**Published:** 2019-12-12

**Authors:** Ji Hui Neo, Siao Ting Teo, Chiew Lan Lee, Cong Cong Cai

**Affiliations:** 1 NG Teng Fong General Hospital, National University Health System, Singapore

**Keywords:** Case report, Frozen shoulder, Telerehabilitation

## Abstract

**Background and objectives:**

Frozen shoulder is a common musculoskeletal condition. Telerehabilitation has seen emerging use in a variety of conditions. This case report aims to investigate the feasibility of adopting telerehabilitation in treating frozen shoulder.

**Case presentation:**

A 43-year old female presented with frozen shoulder of insidious onset. She underwent four sessions of physiotherapy. Sessions two and three were telerehabilitation sessions; the initial and final sessions were conducted in-person.

**Results:**

The subject was compliant with all exercises prescribed during her rehabilitation and achieved all rehabilitation goals in four sessions. She was then discharged from physiotherapy.

**Conclusion:**

Telerehabilitation is feasible in treating frozen shoulder. One barrier to implementation of telerehabilitation includes the lack of technical skills and knowledge despite the high prevalence of technology in today's society. Telerehabilitation increases accessibility and ease of rehabilitation. Telerehabilitation can be considered for segments of the population that are most inclined to use technology.

Frozen shoulder is a common shoulder condition. Recently it has been defined, through consensus, as a condition where both active and passive shoulder motion are restricted without significant imaging findings (Zuckerman & Rokito, 2011). The prevalence of frozen shoulder has been reported to be between 2-10% in the general population (Ramirez, 2019; Walker-Bone, Palmer, Reading, Coggon, & Cooper, 2004; Zreik, Malik, & Charalambous, 2016). Middle-aged women from 40 to 60 years old as well as the diabetic population are at higher risk of developing this condition (Wong & Tan, 2010; Zreik et al., 2016).

Frozen shoulder has been classically divided into three overlapping phases – painful, stiffness (freezing) and recovery (thawing) (Manske & Prohaska, 2008; Ramirez, 2019). While originally thought to be self-limiting with resolution within two years, more recent evidence demonstrating unresolving stiffness has resulted in the re-emergence of the original four-stage classification put forth by Neviaser and Neviaser (1987) with stage four showing marked restriction in range.

The literature remains equivocal between operative and conservative management with studies noting that choice of treatment was largely based on the physician's training and experience rather than evidence (Georgiannos, Markopoulos, Devetzi, & Bisbinas, 2017; Kwaees & Charalambous, 2014). However, physiotherapy is currently the first line and preferred form of conservative treatment according to a survey done in the United Kingdom amongst shoulder and elbow surgeons (Kwaees & Charalambous, 2014).

An emerging method of delivering rehabilitation to patients is through telerehabilitation. A subset of telemedicine, telerehabilitation is the use of telecommunications technology to provide rehabilitation services at a distance (Russell, 2007). Though it only has a short history, telerehabilitation has been gaining traction as a cost-effective method of patient treatment in developed countries (Akiyama & Yoo, 2016). In recent systematic reviews, telerehabilitation has been shown to be comparable to standard practice in improving physical function and pain in the treatment of musculoskeletal conditions (Cottrell, Galea, O'Leary, Hill, & Russell, 2017; Grona et al., 2018). Telerehabilitation is also associated with improved compliance, functional outcomes and reduced travel times (Kruse et al., 2017).

To date, most studies on telerehabilitation focus mainly on postoperative conditions (Pastora-Bernal, Martin-Valero, Baron-Lopez, Moyano, & Estebanez-Perez, 2018; van Egmond et al., 2018), stroke (Tchero, Tabue Teguo, Lannuzel, & Rusch, 2018) or cardiac rehabilitation (Peretti, Amenta, Tayebati, Nittari, & Mahdi, 2017). Systematic reviews on the use of tele-rehabilitation in musculoskeletal conditions have mainly included studies investigating the spine or postoperative populations (Cottrell et al., 2017; Grona et al., 2018). While frozen shoulder is not uncommon, to the best of our knowledge, no study exists for treatment of frozen shoulder with telerehabilitation.

In Singapore, the use of telerehabilitation has been investigated in the stroke population (Koh et al., 2015). A sub-analysis of the barriers and facilitators in telerehabilitation indicated that telerehabilitation was seen as a more affordable and accessible form of treatment by patients, and a way to improve treatment provision by the therapists (Tyagi et al., 2018).

Hence, this case report aims to establish the feasibility of using telerehabilitation in the treatment of frozen shoulder.

## METHODS

A single retrospective case was used to investigate the objectives of this study. This case report adhered to the CARE reporting guidelines (Riley et al., 2017).

### TELEREHABILITATION SYSTEM

The system used was Home-Rehab (T-Rehab, 2018) which is a telerehabilitation system that includes the following components: (1) live video conferencing, (2) patient demonstration of exercises with real time feedback from the therapist, (3) video recording of exercises for physiotherapist follow-up; (4) generation of a dashboard of exercise compliance and exercise statistics; and (5) remote selection of exercises and dosage.

The physiotherapist (ST) involved in the telerehabilitation pilot had received prior training by the vendor on usage of both the therapist and patient version of the applications as well as the hardware. This training involves (1) navigating the user interface and learning how to prescribe exercises through the application; (2) reading and analysing the recorded rehabilitation data; and (3) usage of features as aforementioned which includes videoconferencing and real-time demonstration of exercises by the therapist/patient and subsequent feedback.

### THE SET-UP

**Figure 1.**
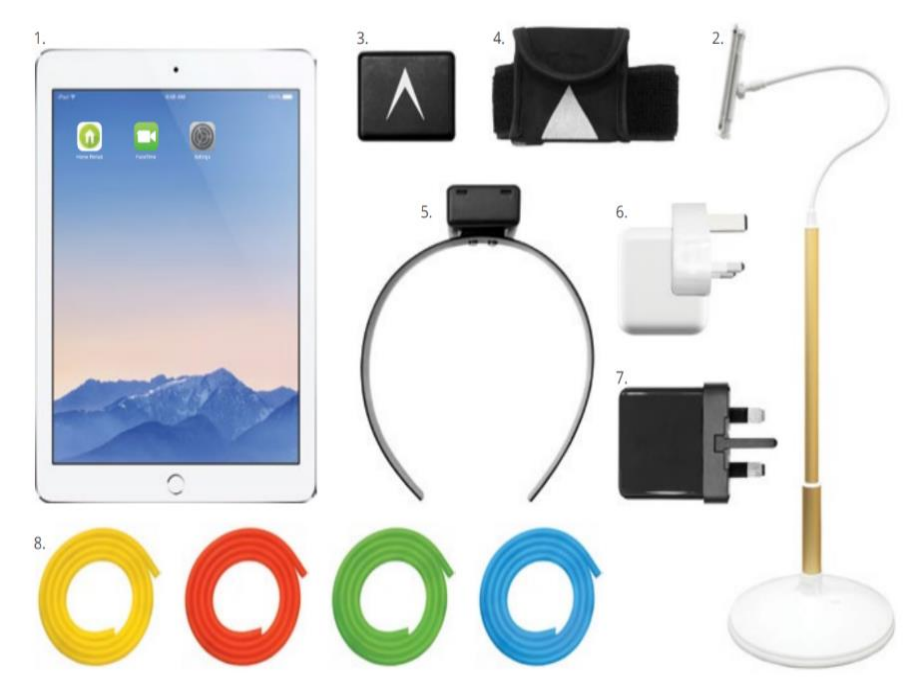
The telerehabilitation kit. Items: (1) iPad; (2) iPad Stand; (3) Limb Sensor; (4) Limb Sensor case; (5) Neck Sensor; (6) iPad charger kit; (7) Sensor charger kit; (8) Resistance tubes: (a) Yellow (Easy), (b) Red (Moderate), (c) Green (Intermediate), (d) Blue (Difficult).

A telerehabilitation kit consisting of the following items was loaned to the patient for the entirety of her rehabilitation period. The kit contains an iPad, an iPad stand, a limb sensor, a limb sensor pouch, a neck sensor, an iPad charger kit and a sensor charger kit. The vendor also provided resistance tubes of varying strengths. The components of the telerehabilitation kit are visually represented below (Figure 1).

**Figure 2.**
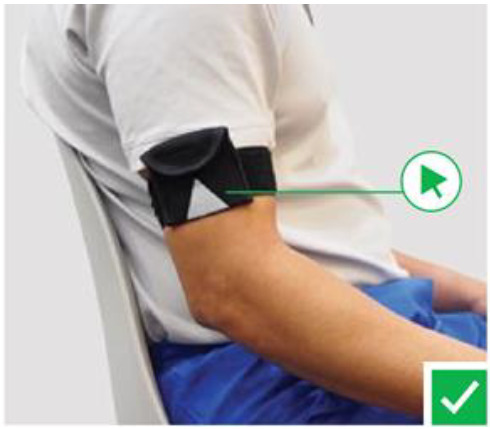
Limb sensor.

**Figure 3.**
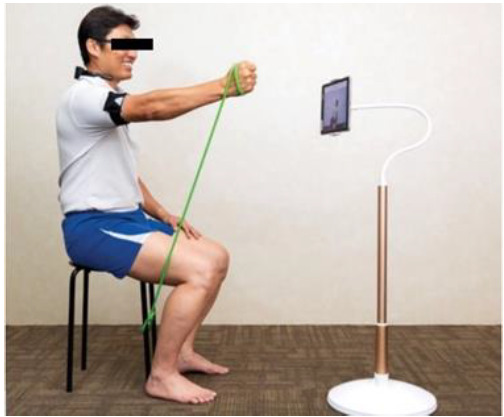
Final set-up (example).

The patient donned one sensor for the upper limb. This sensor was strapped to the mid-portion of the proximal left/right arm (Figure 2). The final set up is shown in Figure 3.

## THE TELEREHABILITATION PROCESS

The patient gave informed written consent to participate in the telerehabilitation programme. Ethics review was not required for a single case report.

During the initial session a complete assessment was conducted. This included measurement of baseline parameters. Following this, the patient was taught exercises and stretches to continue at home while awaiting commencement of the first telerehabilitation session.

The vendor then liaised with the patient to install the system at her home. The patient received training on how to don the straps and sensor prior to commencing the rehabilitation exercises.

The subject had two follow-up telerehabilitation sessions, each a fortnight apart. Every session included a video call between the therapist and subject. The video call included assessment of outcome measures and also verbal feedback from the subject on the exercise programme. After each session, the physiotherapist adjusted the exercises to facilitate maximum patient progression. Each telerehabilitation session was 20 minutes long. All exercises were encouraged to be done thrice daily unless otherwise mentioned.

Once the patient had achieved specific goals and outcomes, the final session was an in-person consultation in the clinic for reassessment and discharge from rehabilitation services.

## THE STUDY

### PARTICIPANT RECRUITMENT

Our subject was recruited from a musculoskeletal specialist outpatient clinic in a tertiary hospital in Singapore. Inclusion criteria for the telerehabilitation programme included (1) a diagnosis of frozen shoulder from the orthopaedic department; (2) reduced shoulder active and passive range of motion; and (3) no previous surgery on the involved upper limb.

### OUTCOMES

Key outcomes were assessed at each session. Objective measures of forward flexion, abduction, and external rotation were measured actively and passively with a goniometer. Internal rotation range was measured as the level of the spinous process that the patient could reach with her thumb going up the back. The Numeric Pain Rating Scale (NPRS) was used to assess pain levels.

### TREATMENT

The subject in our study was a 43-year-old, right-handed female who presented to the physiotherapy outpatient clinic with a diagnosis of frozen shoulder. She had no significant past medical history or comorbidities. This was her first occurrence of frozen shoulder.

Initial subjective examination revealed that the patient was a homemaker and she developed insidious onset of left shoulder stiffness and pain six months earlier with no improvement prior to seeking medical care. Her aggravating factors included; (1) shoulder elevation (NPRS 7/10); and (2) placing her hand behind her back (NPRS 7/10). Her main easing factor was to avoid the abovementioned aggravating activities to which she reported a pain score of zero on the NPRS.

During each session, the physiotherapist taught exercises aimed at progressively increasing strength and reducing pain. The physiotherapist determined the exercise dosage after assessment of the patient. Details of the exercises prescribed in each session as well as each session type are presented in Table 1. The patient was instructed to perform all exercises below pain threshold on a thrice-daily basis using the telerehabilitation system.

**Table 1.** Exercise Prescription

Session No		Session 1	Session 2	Session 3	Session 4
**Session Type**		In-person	Telerehabilitation	Telerehabilitation	In-person
**Exercise Type**	**Strengthening**	-	Flexion with yellow RB to 180° × 20 reps	Flexion with yellow RB to 180° × 25 reps	Flexion with yellow RB to 180° × 25 reps
		-	External Rotation with yellow RB+ to 50° × 20 reps	External rotation with yellow RB to 50° at 30° abduction × 20 reps	External rotation with yellow RB to 50° at 30° abduction × 20 reps
	**Stretching**	Posterior Capsule stretch 30s × 3	Posterior Capsule stretch 30s × 3	Inferior capsule stretch 20s × 3	Inferior capsule stretch 20s × 3
			Inferior Capsule Stretch 20s × 3 reps	Pectoral stretch against wall with arm abducted 90°	Pectoral stretch against wall with arm abducted 90°
	**Range of Motion**	Pole-assisted shoulder abduction to 130° 10 reps × 3 sets	Pole-assisted shoulder abduction to 180° 10 reps × 3 sets	Pole-assisted shoulder abduction to 180° 10 × 3 sets	Shoulder abduction to 180° 10 × 3 sets
		Pole-assisted forward flexion to 150° 10 reps × 3 sets	Pole-assisted shoulder forward flexion to 180° 10 reps × 3 sets	Pole-assisted shoulder external rotation to 50° 20 reps	Shoulder external rotation to 50° 20 reps
		Pole-assisted external rotation to 50° 10 reps × 3 sets	Pole-assisted shoulder external rotation to 50° 10 reps × 3 sets	Internal rotation with towel 20 reps	Internal rotation with towel 20 reps

+ Resistance band

The patient was discharged from rehabilitation on the fourth session as she had achieved full range of motion and was pain-free.

## RESULTS

Overall, the subject achieved her goals of full range of motion with improving internal rotation range (measured as hand behind back) over a period of nine weeks. Results are detailed in Table 2.

**Table 2.** Outcome Measures

Measurements	Session 1	Session 2	Session 3	Session 4
Forward flexion (°)	135	180	180	180
Abduction (°)	115	180	180	180
Left shoulder Internal Rotation (Spinous Process Level)	T12	L1	T9	T5
Hands behind back (NPRS score)	7	4	0	0
Shoulder Flexion (NPRS score)	7	6	3	0

### RANGE

The greatest improvement in range for forward flexion and abduction was seen between session one and session two. Data records showed the subject to have done the exercises more than thrice daily instead of the recommended thrice daily prescription. Full range for both forward flexion and abduction was achieved by the second session while internal rotation improved continuously up until the last session.

### PAIN

Pain scores decreased from seven to zero points by session three when placing hands behind her back and to zero by the final session. The mild pain in shoulder flexion recorded during session three resolved by the time the patient returned for her final in-person session.

## DISCUSSION

Overall, the patient showed good improvement and compliance, not only with the rehabilitation programme but also with the use of the telerehabilitation equipment. Previous literature has suggested that physical touch is an important component of patients' experience of physiotherapy. Our results demonstrate that this is not necessarily essential to success as the patient still managed to achieve her goals (Jensen, Shepard, & Hack, 1990). Certainly, there was not a complete lack of physical interaction, as the first and last sessions were conducted on an in-person basis.

Our patient presented with a six-month history of frozen shoulder. She may have been in transition from the frozen to the thawing stage, which might have facilitated rapid recovery over a total period of nine weeks (Neviaser & Neviaser, 1987). She demonstrated marked improvement in range of motion between the first and second sessions. However, another factor that might have facilitated these outcomes may have been her exercise frequency of three times a day, doing up to 90 repetitions per range of motion exercise in the initial phase of rehabilitation. This is a higher volume of exercise than many individuals would or could perform, and was at variance with the planned rehabilitation protocol. Our results agree with established literature that exercises or gentle stretching done within pain limits may be more effective compared to intensive therapy in frozen shoulder (Diercks & Stevens, 2004; Georgiannos et al., 2017).

The fact that the patient was able to perform her exercise sessions more than three times a day suggests that that she was highly motivated to improve her condition. The accessibility of the telerehabilitation set-up, or what we might term 'semi-supervised therapy', to guide her along in her rehabilitation programme in the convenience of her home might have facilitated her high level of exercise adherence.

Whilst the benefits of telerehabilitation are far-ranging, telerehabilitation does have limitations. A secondary study investigating the factors influencing application of telerehabilitation amongst stroke patients in the local context revealed specific barriers to participation in telerehabilitation including equipment setup, patient assessment, and problems with application use (Tyagi et al., 2018).

National statistics show that 97% of households in Singapore had access to the internet and broadband in 2018, and 74% have used a computer in some fashion. Despite the pervasiveness of technology in residents' homes, 77% cited a lack of skills and knowledge as the main reason for not using the internet. A second factor cited was being too old to learn (14%) (Infocomm Media Development Authority, 2018).

Although telerehabilitation is not novel in Singapore, entrenched traditional attitudes might constitute a challenge to maximising the potential of telerehabilitation as a vehicle of healthcare delivery.

## CONCLUSION

Telerehabilitation as utilised and reported here appeared to be feasible and convenient for the middle-aged patient with good outcomes. We hesitate to generalise the results to all age groups or persons with varying experiences with technology. Telerehabilitation trials involving not only quantitative measures but also qualitative feedback from patients of different age groups and technology skills are needed to determine whether telerehabilitation can provide quality healthcare on a significant scale for patients with frozen shoulders.
